# The Impact of Age on Response to Infection in *Drosophila*

**DOI:** 10.3390/microorganisms9050958

**Published:** 2021-04-29

**Authors:** Noah Sciambra, Stanislava Chtarbanova

**Affiliations:** Department of Biological Sciences, University of Alabama, Tuscaloosa, AL 35487, USA; nmsciambra@crimson.ua.edu

**Keywords:** *Drosophila*, aging, innate immunity, infection

## Abstract

This review outlines the known cellular pathways and mechanisms involved in *Drosophila* age-dependent immunity to pathogenic microorganisms such as bacteria and fungi. We discuss the implication of host signaling pathways such as the Toll, Immune Deficiency (IMD), Janus kinase signal transducer and activator of transcription (JAK/STAT), and Insulin/Insulin Growth Factor/Target of Rapamycin (IIS/TOR) on immune function with aging. Additionally, we review the effects that factors such as sexual dimorphism, environmental stress, and cellular physiology exert on age-dependent immunity in *Drosophila*. We discuss potential tradeoffs between heightened immune function and longevity in the absence of infection, and we provide detailed tables outlining the various assays and pathogens used in the cited studies, as well as the age, sex, and strains of *Drosophila* used. We also discuss the overlapping effects these pathways and mechanisms have on one another. We highlight the great utility of *Drosophila* as a model organism and the importance of a greater focus on age-dependent antiviral immunity for future studies.

## 1. Introduction

The common fruit fly, *Drosophila melanogaster*, with its short lifespan, low cost of culture, and potent conserved innate immune defenses against a variety of microorganisms, serves as an excellent model for investigating the consequences of immunosenescence, a conserved process characterized by the progressive decline of the immune system’s function with age [1,2,3,4]. In humans, immunosenescence is associated with a decreased ability to defend against infections, resulting in significant morbidity and mortality among the elderly [5,6,7,8]. Despite considerable progress made towards our understanding of immunosenescence, the genetic and molecular mechanisms underlying age-dependent responses to immune challenges are areas of ongoing research. Specifically, the interplay between aging and innate immunity, which represents the first line of defense against microbial invaders, is less well understood, and often falls behind studies of the aging adaptive immune system [9]. Individuals aged 65 and older currently outnumber children under 5 globally [10] and, thus, a deeper understanding of the mechanisms underlying immunosenescence has become exceedingly important. A vast repertoire of genetic and genomic tools is available to *Drosophila* researchers, and the high degree of genetic homology between *Drosophila* and humans [11] makes the modeling of human diseases and immunosenescence in *Drosophila* highly translatable [12].

*Drosophila* lack an adaptive immune response but do have conserved pathways underlying innate immunity, which, in mammalian systems, play a key role in the age-dependent response to infections [13,14]. The Toll and Immune deficiency (IMD) pathways in *Drosophila* are nuclear factor kappa B (NF-κB) pathways with similarities to mammalian Toll-like receptor/interleukin (IL)-1 receptor and tumor necrosis factor receptor (TNFR) pathways, respectively. In response to fungal and bacterial infection, activation of Toll and IMD pathways leads to the transcription of downstream antimicrobial peptides (AMPs) genes (reviewed in [15]). Notably, the expression of several AMP genes increases with age [16,17,18]. The Janus kinase/signal transducer and activator of transcription (JAK/STAT) pathway, which, in mammals, is the main signaling downstream of cytokines and their receptors [19], is involved in response to *Drosophila* C Virus (DCV) infection in *Drosophila* [20]. This pathway is also activated in response to bacterial pathogens and is especially important in maintaining homeostasis in the gut, where dysbiosis exacerbates with age [21,22,23,24,25]. The Insulin/Insulin Growth Factor signaling pathway (IIS) which, together with the Target of Rapamycin (TOR) signaling pathway forms the IIS/TOR network, regulates autophagy, detoxification, and protein synthesis in *Drosophila*. Perhaps most importantly, the IIS/TOR pathway is key to lifespan determination in *Drosophila* (reviewed in [26]), and it has a linked function in *Drosophila* immunity due to its interaction with the Toll, IMD, and JAK/STAT pathways in *Drosophila* [27,28,29,30,31]. The Forkhead box, sub-group O transcription factor in *Drosophila* (dFOXO), a downstream target of the IIS/TOR pathway, binds to the promoter region of the AMP gene *Drosomycin* and induces its expression in response to starvation [27]. In *Drosophila*, dFOXO is required for defense against viral pathogens such as the Cricket paralysis virus (CrPV) and the Flock House virus (FHV) [32], and it plays a role in intestinal immunity to the bacterial pathogen *Serratia marcescens* (*S. marcescens)* [33].

**Figure 1:**
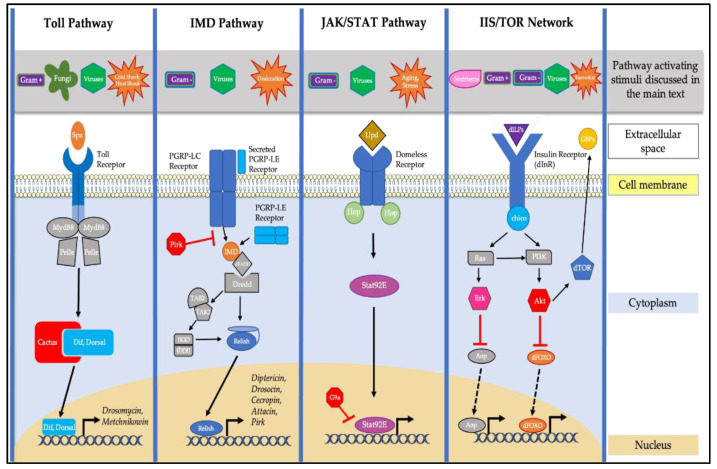
Pathways in *Drosophila* involved in aging innate immunity. The Toll pathway is traditionally activated by Gram-positive bacteria and fungi, and it is also implicated in the response to viral infection, heat shock, and cold shock stresses. Toll activation, via its ligand Spätzle (Spz), leads the degradation of the Cactus inhibitor and subsequent nuclear localization of the NF-κB transcription factors Dif and Dorsal. This leads to the transcription of antimicrobial genes such as *Drosomycin* and *Metchnikowin*. The Immune deficiency (IMD) pathway is primarily activated by Gram-negative bacteria, and is also implicated in the response to viral infection and desiccation stress. Activation of the IMD pathway leads to the nuclear localization of the NF-κB transcription factor Relish and subsequent transcription of genes, including *Diptericin*, *Drosocin*, *Cecropin*, *Attacin,* and *Pirk*. The Janus kinase/signal transducer and activator of transcription (JAK/STAT) pathway is activated via Unpaired (Upd) cytokine detection in response to viral and bacterial infection, aging, and stress. This leads to the nuclear localization of the *Drosophila* Stat92E factor and subsequent activation of transcription. The *Drosophila* Insulin/Insulin Growth Factor/Target of Rapamycin (IIS/TOR) signaling network regulates growth and nutrition via binding of insulin-like peptides (dILPs) to the insulin receptor (dInR). There is a singular insulin substrate, Chico, that induces two downstream signaling cascades, one via the Akt1 kinase and the other via the extracellular signal-regulated kinase (ERK). The IIS/TOR network is impacted by viral (dFOXO specifically), Gram-positive, and Gram-negative bacterial infections, as well as starvation stress. Activation of dInR subsequently affects the activity of the Target of Rapamycin (dTOR) factor, as well as the dFOXO transcription factor.

In addition to these distinct cellular signaling pathways (Figure 1), other factors such as sexual dimorphism, environmental stress, and phagocytic efficacy affect *Drosophila* immunity in an age-dependent fashion. This review will focus on these key pathways and factors, as well as the relevant research assays involved in the study of age-dependent immune responses in *Drosophila.*

## 2. Pathways Affecting Age-Dependent Immunity in *Drosophila*

### 2.1. The Role of the Toll and IMD Pathways in Drosophila Age-Dependent Immunity

*Drosophila* have an inducible antimicrobial immune response that combats pathogenic infections with bacteria and fungi. This response is characterized by the induction of antimicrobial peptides (AMPs) [34], which is controlled by the nuclear factor-κB (NF-κB) family of transcription factors (TFs). NF-κB TFs such as Dorsal (dl), Dif, and Relish are activated through two separate signaling cascades: The Toll and IMD pathways [35,36]. The Toll pathway is primarily activated in response to Gram-positive bacterial and fungal pathogens [35,37], while the IMD pathway is mostly activated in response to Gram-negative bacteria [38,39,40,41]. These conserved innate immune pathways undergo age-dependent changes in function and gene expression. For example, *Drosophila* naturally display increased expression of AMP genes with age [16,17,18,42,43]. Elevated AMP expression typically allows for a more persistent induction of innate immune responses following infection with bacterial pathogens [18]. Some may hypothesize that heightened AMP expression in older individuals allows aged *Drosophila* to respond more efficiently to infection, but studies suggest the opposite. Older flies exposed to both live and killed bacteria (mixture of *Escherichia coli* (*E.coli*) and *Micrococcus luteus* (*M. luteus*)) induce less *Diptericin*, an AMP primarily induced via the IMD pathway, after a heat-killed bacterial jab than younger flies treated simultaneously. Despite the progressive upregulation of AMPs with age, the intrinsic capacity of older flies to effectively induce AMPs and defend against infection is shown to decline with age in *Drosophila* [18].

Age-dependent decline in immune function is conserved across species [3], but there are studies in which aged *Drosophila* display a lower bacterial load than younger *Drosophila* after infection. For example, Khan and Prasad [44] found that, following *S. marcescens* infection, a 13-day-old (aged) LH laboratory population of *Drosophila* displayed lower bacterial loads than younger flies (3 and 8 days old, respectively). However, this study did not include infection survival assays, and studies that do include infection survival assays with long-lived (~80 days median survival) *Drosophila*, such as *chico* null mutants, have found that survival to bacterial infection is not linked to AMP gene expression [45]. Additionally, there are several examples linking overactivation of *Drosophila* NF-κB pathways and over-expression of AMPs with reduction in lifespan [16,46,47]. Kounatidis and colleagues found that brain-specific knockdown of *Relish* increased *Drosophila* lifespan, while overexpression of the AMP genes *AttacinC*, *Drosocin* and *CecropinA1* in neural tissue reduced lifespan [16]. Badinloo and colleagues showed that ubiquitous overexpression of the AMPs *AttacinA*, *Metchnikowin*, *CecropinA1* and *Defensin* resulted in reduced lifespan [47]. In another example, Fabian and colleagues found that *Drosophila* longevity was increased following ubiquitous RNAi knockdown of the genes encoding for Toll (*Tl*) receptor and its ligand Spätzle (*spz*). *Drosophila* longevity decreased ~50% following ubiquitous RNAi knockdown of the Toll pathway inhibitor *cactus* (resulting in overactivation of Toll signaling). The same study also found that long-lived (median lifespan of 62.5–72 days) *Drosophila* caught in a peach orchard in Michigan displayed increased expression of the AMP genes *Drosomycin*, *AttacinA*, and *Diptericin* at a young age (5–6 days old), and decreased expression of these AMP genes at an older age (25–26 days old) following *Erwinia carotovora carotovora* (*Ecc15*) infection, in comparison to a control line [46].

Thus, it is apparent that NF-κB signaling, the ability to defend against infection, and longevity are interconnected in *Drosophila*. The importance of NF-κB signaling at older age from the Fabian et al., [46] study is in support of earlier findings showing that *Rel^E20^* and *Rel^E38^ Relish* null mutations completely eliminate the improved ability of flies over-expressing the intracellular receptor PGRP-LE to defend against *Pseudomonas aeruginosa (P. aeruginosa*) infection [48]. Additionally, although fat body-specific over-expression of PGRP-LE offered enhanced pathogen resistance in both young (7 day-old) and older (40 day-old) animals, this chronic activation of NF-κB signaling resulted in a shorter lifespan in the absence of infection in comparison to *Drosophila* carrying a *Relish* null mutation [48].

All of these findings suggest a tradeoff between pathways involved in immune signaling and longevity in *Drosophila*. Findings from Sinam and colleagues [49] build upon this trend. The authors compared the lifespan and response to bacterial and fungal pathogens of two *Cytorace* lines derived from hybridization of *Drosophila nasuta nasuta* and *Drosophila nasuta albomicans*, and which were at different stages of evolutionary divergence. They showed that *Cytorace-9* flies failing to upregulate expression of the *Cecropin* gene were more susceptible to infection with the fungal pathogens *Beauveria bassiana* (*B. bassiana*) and *Metarhizium anisopliae* (Table 1) in comparison to *Cytorace-3* flies, which upregulate *Cecropin* expression after infection. *Cytorace-9* flies also showed increased lifespan in the absence of infection when compared to *Cytorace-3* flies. While increased AMP gene expression may offer increased pathogen resistance, especially at a younger age, this heightened immune signaling negatively impacts lifespan in the absence of infection. However, given that survival to bacterial infection was shown to not be linked to AMP gene expression in long-lived *chico Drosophila* mutants [45], this tradeoff between immune signaling and longevity in *Drosophila* is likely more complex than current research suggests [49]. Further research is necessary to fully elucidate the complex nature of the age-dependent increase of AMP expression in *Drosophila* and how it affects lifespan and response to infection.

### 2.2. The Role of the JAK/STAT Pathway in Drosophila Age-Dependent Immunity

The Janus kinase (JAK) signal transducer and activator of transcription (STAT) signaling pathway is induced by the cytokines of the Unpaired family, such as Unpaired 1 (Upd1), Unpaired 2 (Upd2), and Unpaired 3 (Upd3), in response to cellular stress and damage [19,50,51]. The JAK/STAT signaling pathway plays an important role in *Drosophila* adult midgut homeostasis, ensuring continuous renewal of this organ throughout the animal’s lifespan [51]. The Toll pathway does not function in the *Drosophila* gut [52], and the JAK/STAT pathway contributes to gut antimicrobial response through the induction of Drosomycin-like peptides [24]. Given that dysfunction of the intestinal barrier is a hallmark of aging in *Drosophila* [53], and that a functional intestinal barrier during aging is critical to maintaining lifespan [54], it is thus understandable how JAK/STAT signaling may be involved in age-dependent immunity in *Drosophila*.

Intestinal epithelial renewal is a key defense against oral bacterial infection in *Drosophila*, and the JAK/STAT pathway is required for bacterially induced stem cell proliferation in response to infection, stress, or damage [21,22,24,54]. Salazar and colleagues [54] studied the effects of altered expression of the septate junction-specific protein Snakeskin (Ssk), in combination with the JAK/STAT pathway, and observed significant effects on intestinal barrier dysfunction, dysbiosis, lifespan, and gut morphology (Table 2). Decreased expression of intestinal *Ssk* resulted in increased barrier dysfunction, reduced lifespan, elevated expression levels of the AMP genes *Diptericin, Drosocin, Drosomycin,* and *Metchnikowin*, and increased *upd3* mRNA levels. Restoration of *Ssk* expression completely reversed age-related intestinal barrier dysfunction, protected against *S. marcescens* infection, and increased lifespan. Thus, there is a correlation between increased *upd3* expression, an activator of the JAK/STAT pathway, and junctional protein mislocalization before detectable intestinal barrier failure. This also aligns with previous studies demonstrating elevated *upd3* expression in fly mutants for the human CD36 homologue *croquemort* (*crq^KO^*), which is involved in microbial phagocytosis and clearance. *crq^KO^* mutants are short-lived and exhibit premature aging associated with gut hyperplasia [55]. This suggests that proper regulation of the JAK/STAT pathway is central in preventing age-dependent gut dysfunction and protecting against infection.

In addition to its role in age-dependent intestinal barrier homeostasis and defense against oral bacterial infection, the JAK/STAT pathway functions in antiviral immunity, particularly in response to infection with the RNA viruses *Drosophila* C Virus (DCV) and Cricket paralysis virus (CrPV) [20,56], as well as the DNA virus Invertebrate iridescent Virus 6 (IIV-6) [57]. In *Drosophila,* the histone H3 lysine 9 methyltransferase G9a was shown to negatively regulate JAK/STAT pathway activation in response to RNA virus infection in order to limit immunopathology caused by hyperactivation of this pathway [58]. Although *G9a* mutants appear to have increased lifespan in comparison to respective wild-type controls, these flies succumb faster to infection with the RNA viruses FHV, CrPV, DCV and *Drosophila* X virus (DXV) [58]. Fabian and colleagues [46] identified *upd3* as a candidate gene for longevity in *Drosophila* and infected long-lived (median lifespan of 62.5–72 days) wild-caught *Drosophila* from a Michigan peach orchard at 5–6 days of age with DCV. Flies of this long-lived strain injected with DCV survived significantly longer than random-bred control lines of the same population, suggesting that prolonged lifespan may be significantly linked to improved realized antiviral immune response. These findings further complicate the relationship between aging and immunity and the fitness tradeoffs that may arise from favoring expression of genes important for extending lifespan or for defense against infection. Maintaining epithelial junctions may be critical to prolonging lifespan and defending against infection with age, but further research is needed to fully understand the complexity of these systems and what fitness tradeoffs come as a result of age-dependent changes in JAK/STAT signaling.

### 2.3. The Role of the IIS/TOR Network in Drosophila Age-Dependent Immunity

The Insulin/Insulin Growth Factor (IIS) signaling pathway and its linked Target of Rapamycin (TOR) signaling pathway are vital nutritional systems that regulate growth in *Drosophila* [59]. *Drosophila* have one Insulin receptor (dInR), a singular insulin receptor substrate (*chico*), one downstream dFOXO transcription factor, and eight dInR ligands: insulin-like peptides (dILPs) 1–8 [60,61]. Activation of dInR induces two possible downstream signaling cascades, one via the kinase Akt1 and the other via the mitogen activated protein (MAP) kinase Rolled, also known as extracellular signal-regulated kinase (ERK). Akt1 negatively regulates dFOXO and positively regulates TOR [62,63]. Rolled (ERK) activation is implicated in cellular growth and insulin sensitivity [64,65].

IIS signaling pathway functionality is directly linked to longevity and immunity in *Drosophila*. The Toll, IMD, and JAK/STAT pathways interact with the IIS signaling pathway in the *Drosophila* fat body to regulate metabolism, growth, and immunity. dFOXO regulates AMP gene expression [27], and the Toll NF-κB transcription factor Dif can inhibit insulin signaling [28]. Additionally, IIS and subsequent Rolled/MAPK induction can activate the fly *poor Imd response upon knock-in* (*Pirk*), a gene encoding for a negative regulator of the IMD pathway [64,66,67,68,69]. Furthermore, the *Drosophila* fat body signals with insulin producing cells (IPCs) located in the *Drosophila* brain to communicate nutrient status and the release of insulin-like peptides (dILPs). This is accomplished via secretion of another cytokine from the Unpaired family, Unpaired 2 (Upd2), a ligand for the JAK/STAT pathway, when *Drosophila* are in a fed state [70]. This all emphasizes the possible role of the IIS signaling pathway in age-dependent immunity, given that the Toll, IMD, and JAK/STAT pathways have significant roles in *Drosophila* aging and immunity that have already been discussed.

Reduced IIS signaling extends lifespan [71,72], slows or delays age-dependent organ degeneration [73], and improves climbing ability in *Drosophila* [74]. A study from Ueda and colleagues found that increased expression of the miRNA *miR-305*, which usually decreases with age, accelerates aging phenotypes in *Drosophila*. Notably, mRNAs for insulin-like peptides also increased, along with *miR*-305 expression, further emphasizing the link between reduced IIS/TOR signaling and increased lifespan [75]. Growth-Blocking Peptides (GBPs) are produced as a direct result of TOR signaling. Reduced GBP expression reduces *Drosophila* growth rate and body size [76]. Genetic analyses from Sung and Shears [77] have linked a previously uncharacterized G Protein Coupled Receptor (GPCR), Methuselah-like receptor-10 (Mthl10), as a binding protein for GBP in *Drosophila*. As in other studies where reduced IIS/TOR signaling increased lifespan, *Mthl10* knockdown in *Drosophila* also increased lifespan. This increased lifespan as a result of *Mthl10* knockdown also affected pathogen defense against infection. *Mthl10* knockdown increased mortality in adult *Drosophila* after infection with *M. luteus* [77]. This suggests a possible fitness tradeoff between increased longevity and age-dependent resistance to infection via IIS/TOR signaling similar to the tradeoffs seen in the Toll and IMD pathways.

In addition to its known role in regulating lifespan across numerous species, the IIS/TOR pathway also regulates homeostasis of the *Drosophila* lymph gland, a larval hematopoietic organ important for the generation of blood cell progenitors [78]. Despite the disappearance of the *Drosophila* lymph gland after the larval stage of development [79], genetic analysis of *Drosophila* infected with *E. coli* at 4 weeks of age still found genes involved with the IIS/TOR pathway to be significantly associated with bacterial clearance at older age [80]. Additionally, mutations in *chico*, a *Drosophila* insulin receptor substrate, extended lifespan and increased survival of mutant flies following *E. coli* and *Photorhabdus luminescens* (*P. luminescens*) infection [81]. Following *E. coli* and *P. luminescens* infection, *chico* mutants survived bacterial infection better than control flies, and displayed significantly lower amounts of bacterial cells at 3 and 16 h post-infection. However, *chico* mutants displayed higher bacterial loads at 30 h post-infection in response to *E. coli* infection, but not *P. luminescens* infection. These mutants also showed reduced transcription of the AMP genes *Diptericin*, *CecropinA1*, and *Drosomycin*, although pathogen- and time point post infection-based variation was observed [81]. These results suggest a link to age-dependent immunity for the IIS/TOR pathway.

Furthermore, the downstream target of the IIS/TOR signaling pathways, dFOXO, is essential to both intestinal immunity against *S. marcescens* and defense against viral pathogens [32,33]. In comparison to *wild-type* controls, *dFOXO* mutants die faster after oral infection with *S. marcescens*, and male *dFOXO* mutants accumulate higher bacterial loads [33]. *dFOXO* null mutants are also deficient in fighting off infection from both CrPV and FHV [32]. Additionally, dFOXO activity increases in response to virus infection and activated dFOXO can decrease viral load following infection [32], displaying a role for the linked IIS/TOR pathway in *Drosophila* innate immunity (Table 3). Interestingly, the number of FOXO-bound genes significantly decreases with age in female *Drosophila* (comparing 2-week-old and 5-week-old *w^1118^* control flies), and many FOXO-targeted genes have altered transcription levels with age [82]. However, it is also notable that the FOXO transcription factor is not only influenced by the IIS/TOR pathway, and many of the observed age-dependent changes in FOXO DNA binding were independent of the Insulin signaling pathway [82]. With its role both within and independent of the IIS/TOR signaling pathway, FOXO will be a transcription factor of very high interest for future studies investigating age-dependent immunity in *Drosophila*.

The IIS/TOR pathway appears to be significantly involved in age-dependent immunity and the control of lifespan in *Drosophila*, but further research is needed to understand the complexity of these systems and the fitness tradeoffs that may come with reduced IIS/TOR signaling. Additionally, the FOXO transcription factor, its diverse mechanisms in transcriptional regulation, and its altered targeting and function related to the IIS/TOR pathway with age requires further research into the dynamics of FOXO’s roles in both aging and immunity.

## 3. Other Factors Affecting *Drosophila* Age-Dependent Immunity

There are various environmental, genetic, and physiological factors that can affect age-dependent immunity in *Drosophila*. One of these factors is sex [83,84]. The causes of immunosenescence can vary between sexes. Both males and females become more susceptible to infection with age, but the cause of immunosenescence can vary between sexes. Following infection with the fungus *B. bassiana*, age-dependent decline in immune function for males results from barrier defense deterioration, while, in females, it results from systemic senescence of immune defenses [85]. However, Khan and Prasad found that sex did not affect bacterial load levels following *S. marcescens* infection [44]. The true significance of sex in respect to age-dependent immunity in *Drosophila* specifically is yet to be determined but, given the established profound differences in immune response between males and females [84], it can be expected that future immunosenescence research may display significant sexually dimorphic results.

Environmental factors, which have sexually dimorphic effects, also have a significant impact on age-dependent immunity in *Drosophila*. Cold stress, subjecting *Drosophila* to periods of extreme low temperatures, has beneficial effects on aging, lifespan, and resistance to stresses such as severe temperature exposure and *B. bassiana* infection [86,87,88]. Additionally, these environmental stresses have different effects on males and females. Cold stress in male *Drosophila* increases longevity when applied before 4 weeks of age, while providing no positive effect in female *Drosophila* at any age. Meanwhile, cold stress can increase heat resistance ability at 6 weeks of age in both sexes, no matter when the cold stress was applied. This displays that exposure to environmental stress early in life can improve resistance to environmental stress later in life, regardless of sex. Prior exposure to cold stress increased survival to fungal infection (*B. bassiana*) at 6 weeks of age in both sexes, although males were more significantly affected. These differences in pathogen defense between sexes at old age highlights the significance of both environmental stress and sexual dimorphism on age-dependent immunity [87].

Other environmental factors, such as desiccation, also affect age-dependent immunity in *Drosophila*. Desiccated flies that were kept in empty vials without food for 2 h displayed an increased susceptibility to *Ecc15* infection [89]. In the same study, the authors showed that desiccated flies allowed to recover prior to *Ecc15* infection display higher survival rates than flies not desiccated prior to infection. This suggests that being under significant environmental stress during infection decreases resistance, but that exposure to an environmental stress with allowed recovery prior to infection increases resistance. This short desiccation period elevates levels of peptidoglycan recognition protein-LC (PGRP-LC) expression in Malpighian tubules (the equivalent of the kidney) and increases induction of the AMP genes *CecropinA2*, *CecropinC*, *AttacinD*, *Diptericin*, *Defensin*, *and Metchnikowin* [89]. This further emphasizes the impact of environmental factors on age-dependent immunity in *Drosophila*, and these factors should be accounted for when designing experiments relevant to aging and immunity.

In addition to environmental and physiological factors, the functionality of basic cellular mechanisms can have significant impacts on age-dependent immunity in *Drosophila*. Findings from Horn and colleagues highlight the effects of *Drosophila* phagocytic efficiency, which significantly declines with age [90]. Notably, the rate of *Escherichia coli* engulfment after infection does not decline, but the functional clearance of phagocytic vesicles after engulfment decreases considerably with age. Four lines from the *Drosophila* Genetic Reference Panel (DGRP) collection [91,92] (lines 359, 389, 437, and 589), with increased or decreased bacterial clearance with age, display the same number of phagocytic events per hemocyte, suggesting that the functional clearance of engulfed bacteria, and not engulfment itself, is an important factor in age-dependent immunity [90] (Table 4).

## 4. Conclusions and Future Directions

There are a variety of cellular pathways and mechanisms, as well as environmental and physiological factors, that affect the ability of aged organisms to respond to infection. Additionally, many of these pathways and factors appear to have tradeoff effects on immune function and lifespan. Toll and IMD pathways activate the NF-κB TFs in *Drosophila* and subsequent AMP expression [15,34], and *Drosophila* naturally display higher AMP expression at an older age [16,17,18,42,43,46,47]. The Toll and IMD pathways are critical to survival against bacterial, fungal, and viral infection [35,37,38,93,94,95,96], but overactivation of these pathways has been shown to decrease lifespan in the absence of infection [48]. The JAK/STAT signaling pathway contributes to antimicrobial response and homeostasis in the *Drosophila* gut [51]. Maintaining a functional intestinal barrier is critical to maintaining lifespan, and dysfunction of the intestinal barrier is a hallmark of aging in *Drosophila* [53,54]. Intestinal epithelial renewal is vital for protection against oral bacterial infection, and the JAK/STAT pathway is required for this process [21,22,24,54]. In addition to maintaining gut homeostasis and defending against bacterial infection, the JAK/STAT pathway is also important for defense against viruses such as DCV, CrPV and IIV-6 infection [20,56,57]. *Upd3* has been identified as a candidate for longevity in *Drosophila*, and wild-caught long-lived fly strains survive longer than controls following DCV infection [46]. The IIS/TOR signaling pathways regulate growth, longevity, and defense against infection in *Drosophila* [59]. Reduced IIS/TOR signaling extends lifespan [71,72] and has shown contrasting effects on the ability to respond to bacterial infection in adult *Drosophila* [80,81]. The IIS/TOR network also interacts with the Toll (Dif), IMD (Pirk), and JAK/STAT (Upd2) pathways [28,69,70], further emphasizing its role in *Drosophila* age-dependent immunity. dFOXO, a transcription factor downstream of the IIS/TOR signaling pathways, has age-dependent changes in its DNA binding activity [82], and is important for defense against viral pathogens such as FHV and CrPV [32]. There are significant differences in response to infection by male and female *Drosophila* [84]. Different causes have been identified for the age-dependent decline in anti-fungal immune function between male and female *Drosophila* [85], but this does not appear universal, as another study found no significant differences in bacterial loads between male and female flies [44]. Environmental stressors such as cold stress and desiccation have positive effects on lifespan and resistance to fungal [86,87] and bacterial infection [89]. Additionally, cellular mechanisms such as phagocytic efficiency significantly decline with age following *E. coli* infection [90].

The studies discussed in this review illustrate at least some of the aspects the impact of aging exerts on immune function in *Drosophila*. However, further studies are needed to create a more complete understanding of the age-dependent factors and mechanisms that impact *Drosophila* immune defenses and the fitness tradeoffs between longevity and the ability to defend against infection. Additionally, given the profound impact of viral pathogens on elderly individuals, as illustrated by the ongoing Coronavirus (COVID-19) global pandemic [97,98,99,100] and the relative lack of focus on age-dependent immunity in response to viral infection, there is a great need for further research investigating the age-dependent mechanisms that defend against viral infection specifically. *Drosophila* can serve as a great genetic model to investigate the impact of age on innate immune responses to viral infection.

Despite well-characterized pathways involved in *Drosophila* antiviral immunity, such as antiviral RNAi (reviewed in [101,102]) and cellular processes such as autophagy, apoptosis, and apoptotic body clearance (reviewed in [103]), there is very limited understanding of how these antiviral immune responses are impacted by aging. Interestingly, some of the pathways already outlined in this article, which have been shown to have age-dependent effects on *Drosophila* immunity in other contexts, such as the JAK/STAT, Toll and IMD pathways, are also important for *Drosophila* antiviral immunity. The JAK/STAT pathway has been shown to mediate survival to DCV, CrPV, FHV, and DXV infection [20,56,58]. Toll pathway mutants die more rapidly following DXV infection [93], and Toll pathway genes have been shown to be important for resistance to several RNA viruses (DXV, DCV, FHV, CrPV and Nora Virus) [93,94]. IMD pathway mutants are more susceptible to CrPV and Sindbis Virus (SINV) infection [95,96]. Thus, these pathways should be of high interest in future *Drosophila* immunosenescence research. Additionally, antiviral cellular mechanisms, such as phagocytosis via macrophages, display an age-dependent decline in efficiency [90,104].

Another particular area of future research interest is in the importance of disease tolerance mechanisms in response to viral infection at older age. There are two strategies for organisms to defend against infection: resistance and tolerance. Disease resistance encompasses an organism’s ability to combat and prevent infection by pathogens. Disease tolerance encompasses an organism’s ability to tolerate a given level of infection (pathogen load) and limit negative effects after resistance mechanisms have been compromised (reviewed in [105]). Disease tolerance at older age may be very important for antiviral defense in *Drosophila*. In a recent study, Sheffield and colleagues found that, despite displaying an increased susceptibility to FHV infection, older (30 day-old) wild-type (*OregonR*) flies do not accumulate higher virus titers than their younger (5 day-old) counterparts [106]. Intriguingly, a similar pattern has been observed in humans following infection with severe acute respiratory syndrome coronavirus 2 (SARS-CoV-2), the pathogen responsible for COVID-19. In comparison to younger individuals, older individuals display increased mortality without accumulating a higher virus titer when infected with SARS-CoV-2 [107]. Clearly, there is still much to be discovered regarding immune defenses in both humans and *Drosophila*, especially regarding the impact of age on these immune defenses. *Drosophila* can be a powerful tool in our pursuit to elucidate the intricacies of immune defense pathways and their change in efficiency over time. With the availability of advanced genetic and genomic tools in *Drosophila*, future findings made in this organism could lead to important translational impacts and advancements in healthcare, as well as treatment strategies that lead to improved immune response and increased longevity in aged organisms.

## Figures and Tables

**Table 1:** Studies implicating the Toll and IMD pathways in age-dependent immunity.

Reference	Pathogen(s) Used	Assay(s) Used	Age/Sex of Flies Used in the Study	Altered Genes (Pathways)
Sinam et al., 2016 [49]	*Bacilus subtilis* (Gram+) *Beauveria bassiana* (Fungus) *Enterobacter cloacae* (Gram-) *Metarhizium anisopliae* (Fungus) *Serratia marcescens* (Gram-) *Staphylococcus aureus* (Gram+)	Lifespan assay in the absence of infection Heat stress survival assay (37 °C) Oxidative stress resistance assay Infection survival assay (~10^12^ colony forming units (CFU)/mL concentration for bacteria used in survival assays) Bacterial survival in hemolymph assay (hemolymph extracted 6 h post-infection. ~100 CFU of *S. marcescens* and *S. aureus* incubated with hemolymph for 2 h at 25 °C. 100 µL then plated for bacteria growth at room temperature).	Male and female flies raised at 25 °C separately used for lifespan assay Males and females of unspecified age separately used in heat stress assay, oxidative stress resistance assay, infection survival assay, and microbial load assay	Suggested Toll pathway (*Cecropin* expression measured via RT-qPCR)
Zerofsky et al., 2005 [18]	*Escherichia coli* (Gram-) (Concentration not specified) *Micrococcus luteus* (Gram+) (Concentration not specified)	Infection pricking assay (mixture of *E. coli* and *M. luteus*) Immune response assay after injection (measuring *Diptericin* AMP expression) Fecundity assay after injection (counting daily egg production)	Flies raised at 25 °C Females aged 1, 2, 3, and 4 weeks old used in the infection assay and the immune response assay Virgin females mated to males for three days used in the fecundity assay (injected at 4 d of age and newly laid eggs counted daily)	IMD pathway (*Diptericin* expression after infection as a function of age; fecundity after infection in wild-type flies, hypomorph *imd1-1* mutants, and null *Rel^E38^* mutants)
Fabian et al., 2018 [46]	*Beauveria bassiana* (Fungus) (Concentration not specified) *Drosophila* C Virus (+ssRNA virus) (2 × 10^7^ particles/mL = TCID_50_) *Enterococcus faecalis* (Gram+) (OD_λ = 600 nm_ = 8) *Erwinia carotovora carotovora* (Gram-) (*Ecc15*) (OD_λ = 600 nm_ = 200)	Bacterial clearance assay after infection Lifespan assay following infection	Flies raised at 25 °C Females aged to 5–6 days old and 23–25 days old used in the bacterial clearance assay (only 5–6-day-old females used for *Drosophila* C Virus infection) Males and females raised together from eclosion to death used in the lifespan assay	Toll pathway (ubiquitous RNAi knockdown of *Spätzle*, *Toll*, *cactus*, and *Dif*) JAK/STAT pathway (*Upd3* identified as a candidate gene for longevity)
Libert et al., 2006 [48]	*Enterobacter cloacae* (Gram-) (OD_λ = 600 nm_ = 1–70) *Enterococcus faecalis* (Gram+) (OD_λ = 600 nm_ = 70) *Pseudomonas aeruginosa* (Gram-) (OD_λ = 600 nm_ = 0.004) *Staphylococcus aureus* (Gram+) (OD_λ = 600 nm_ = 0.5)	Infection survival assay Climbing assay in the absence of infection (measured using vertical vial and mechanical stimulation after 20 s) Fecundity assay (eggs counted every 24 h for 7 days after mating) Heat-shock tolerance assay (37 °C) Lifespan assay in the absence of infection	Fly rearing temperature not specified 10-day-old separated males and females used in the infection survival assay 10-day-old flies of unspecified sex were used in the climbing assay and the heat-shock tolerance assay Female flies of unspecified age used in the fecundity assay Separated males and females used in the lifespan assay	IMD pathway (*Rel^E20^* and *Rel^E38^* null mutants, fat body-specific knockdown (*FADD, TAK1* dominant-negative), and overexpression (PGRP-LE) using S106-GS-Gal4)
Libert et al., 2008 [45]	*Pseudomonas aeruginosa* (Gram-) (OD_λ = 600 nm_ = 0.004) *Enterococcus faecalis* (Gram+) (OD_λ = 600 nm_ = 70)	Infection survival assay Lifespan assay in the absence of infection Immune response assay after injection (*Diptericin, Drosomycin,* and *Attacin* expression using RT-PCR)	Flies raised at 25 °C Male and female flies of unspecified age used in the infection survival assay, lifespan assay, and immune response assay	NF-kB pathway (*Diptericin, Drosomycin,* and *Attacin* expression via RT-PCR) Insulin signaling pathway (infection survival assay and lifespan assay using *chico* null mutants)

**Table 2:** Studies implicating the JAK/STAT pathway in age-dependent immunity.

Reference	Pathogen(s) Used	Assay(s) Used	Age/Sex of Flies Used in the Study	Altered Genes (Pathways)	Other Factors Investigated
Salazar et al., 2018 [54]	*Serratia marcescens* (Gram-) (OD_λ = 600 nm_ = 1)	Smurf and smurf reversal assays (flies kept on blue food for 24 h before being scored for blue dye leakage) Bacterial load assay via genomic DNA isolation Starvation resistance assay (25 ug/mL RU486 in water only medium) Triacylglyceride assay (lipids extracted from whole flies) Lifespan assays with and without RU486-mediated transgene induction	Flies raised at 25 °C 10- and 17-day-old female flies used in the bacterial load assay 3-day-old females used in the starvation resistance assay 9-, 13-, and 30-day-old female flies used in fluorescence microscopy and ratios for Ssk, Dlg, Mesh, and Coracle localization	Snakeskin junction protein (knockdown of *Ssk* using RNAi) JAK/STAT (elevated *upd3* expression following depletion of Ssk)	Intestinal junction proteins (Ssk, Dlg, Mesh, and Coracle localization) in Smurf and non-Smurf flies
Fabian et al., 2018 [46]	*Beauveria bassiana* (Fungus) (Concentration not specified) *Drosophila* C Virus (+ssRNA virus) (2 × 10^7^ particles/mL = TCID_50_) *Enterococcus faecalis* (Gram+) (OD_λ = 600 nm_ = 8) *Erwinia carotovora carotovora* (*Ecc15*) (Gram-) (OD_λ = 600 nm_ = 200)	Bacterial clearance assay after infection Lifespan assay following infection	Flies raised at 25 °C Females aged 5–6 and 23–25 days old used in the bacterial clearance assay 1–4-day-old females used for DCV infection Males and females raised together from eclosion to death used in the lifespan assay	JAK/STAT pathway (*upd3* identified as a candidate gene for longevity) Toll pathway (ubiquitous knockdown of *Spätzle*, *Toll*, *cactus*, and *Dif* using RNAi)	

**Table 3:** Studies implicating the IIS/TOR network and dFOXO Transcription factor in age-dependent immunity.

Reference	Pathogen(s) Used	Assay(s) Used	Age/Sex of Flies Used in the Study	Altered Genes (Pathways)	Other Factors Investigated
Felix et al., 2012 [80]	*Escherichia coli* (Gram-) (OD_λ = 600 nm_ = 1, ~5.5 × 10^8^ CFUs/mL)	Bacterial clearance assay after infection Genome Wide Association Study (GWAS) utilizing DGRP lines	Flies raised at 25 °C 1- and 4-week-old flies of unspecified sex were used in the bacterial clearance assay	Insulin signaling/TOR pathway (alterations in *Akt* and *mos* are associated with variation in bacterial clearance ability at older age) IMD pathway (*Relish*, *AttacinA*, *AttacinB*, *AttacinD*, *CecropinB and C*, *DiptericinB*, *PGRP-SD*, *PGRP-LF*, *PGRP-SB1* found to be upregulated with age)	Metabolism (several candidate genes involved in metabolic functions associated with bacterial clearance ability at older age)
McCormack et al., 2016 [81]	*Escherichia coli* (Gram-) (non-pathogenic K12 strain) (100–300 CFU) *Photorhabdus luminescens* (Gram-) (Strain TT01) (100–300 CFU)	Infection survival assay Bacterial load assay after infection (measured at 3, 16, and 30 h post-infection. CFU estimated from the standard curves generated from *E. coli* and *P. luminescens*) Phenoloxidase activity assay after infection (measured at 3 h post-infection. OD measured at 492 nm against a blank control) Phagocytosis estimation assay after infection (measured 1-h post-infection via fluorescent imaging of the fly dorsal surface) Metabolic activity assay after infection (measured at 3- and 18 h post-infection. Absorbance measured at 562 nm and protein concentration of the samples calculated from a standard curve)	Flies raised at 25 °C 7–10-day-old males and females raised together were infected in the infection survival assay, bacterial load assay, phenoloxidase activity assay, phagocytosis estimation assay, and the metabolic activity assay	Insulin-like growth factor signaling (*chico^KG00032^* mutants) IMD pathway (measured *Diptericin* and *Cecropin-A1* gene expression via RT-qPCR in *chico^KG00032^* mutants) Toll pathway (measured *Drosomycin* gene expression via RT-qPCR in *chico^KG00032^* mutants)	
Sung et al., 2018 [77]	*Micrococcus luteus* (Gram+) (Concentrated culture, CFU not listed)	Infection survival assay Lifespan assay in the absence of infection	Flies raised at 25 °C Adult males of unspecified age used in the infection survival assay Females used in the lifespan assay	*Methuselah-like receptor-10* (*Mthl10*) (ubiquitous knockdown of *Mthl10* using RNAi) Insulin-like Peptide (ILP) secretion (ubiquitous knockdown of Growth-Blocking Peptide cytokine with *Actin-Gal4>UAS-GBP^RNAi^* line) NF-kB pathway (measurement of *Metchnikowin* gene expression in *Mthl10* RNAi lines)	
Spellberg et al., 2015 [32]	*Cricket Paralysis Virus (CrPV)* (+ssRNA) (100 and 300 TCID_50_) *Flock House Virus (FHV)* (+ssRNA) (5 * 10^5^ PFU)	Infection survival assay Heat Shock assay in the absence of infection (flies placed at 37 °C for 1h and then incubated at 25 °C for 6h) Cell culture (*Drosophila* S2 cells with inducible constitutively active FOXO plated at 1 × 10^6^ cells per mL)	Fly rearing temperature not specified 5–7-day-old flies of unspecified sex were used in the infection survival assay Male flies of unspecified age were used in the heat shock assay	*dFOXO* null mutants (*FOXO^Δ94^*) used in the infection survival assay Heat shock-mediated knockdown of *dFOXO* using RNAi	
Ueda et al., 2018 [75]	No pathogen used	Lifespan assay in the absence of infection Climbing assay (measured via vertical vial and mechanical stimulation after 6 s) RNA-seq (performed in *miR-305* over-expressing flies), analysis for possible *miR-305* target mRNAs) Immunostaining (targeting poly-ubiquitinated proteins and abnormal protein aggregates)	Flies raised at 28 °C Male adult flies (24 h after eclosion-death) were used in the lifespan assay Male adult flies ranging from 0 to 45 days old were used in the climbing assay.	Insulin-like growth factor signaling (IIS) pathway (RNAseq analysis showed that *tobi*, *dilp2*, and *dilp5* mRNA increased upon *miR-305* over-expression and that mRNA for *dilp6* and *dilp8* decreased upon *miR-305* over-expression). Toll pathway (reported that *miR-305* targets *ModSP* mRNA, which is part of the Toll signaling cascade)	

**Table 4:** Studies implicating other factors in age-dependent immunity.

Reference	Pathogen(s) Used	Assay(s) Used	Age/Sex of Flies Used in the Study	Altered Genes (Pathways)	Other Factors Investigated
Khan et al., 2013 [44]	*Serratia marcescens* (ATCC 13880) (Gram-) (OD_λ = 600 nm_ = 1)	Bacterial load assay	Flies raised at 25 °C 3-, 10-, and 13-day-old males and females raised separately used in the bacterial load assay		Sexual dimorphism (No significant difference in bacterial load by sex) Age-dependent immunity (lower bacterial load in older flies (13 day-old) than younger flies (3 day-old))
Horn et al., 2014 [90]	*Escherichia coli* (Gram-) (heat-killed, 6 × 10^9^ particles/mL diluted 1:6)	Whole-fly fluorescence assay after infection Hemocyte fluorescence assay after infection In-vivo quantitative phagocytosis assay (imaged 90 min after infection) Phagocytic ability assay after infection (measured as the amount of fluorescent *E. coli* per active hemocyte 90 min after infection) Bead engulfment assay (flies infected with fluorescent plastic beads which cannot be broken down) (Alexa Fluor 568-labeled 1-µm beads)	Fly rearing temperature not indicated 1- and 5-week-old females used in the whole-fly fluorescence assay, hemocyte fluorescence assay, and bead engulfment assay Adult virgin female *Canton-S* flies of unspecified age were used in the in-vivo quantitative phagocytosis assay 5–7-day, 35–39-day, 1-week, and 5-week-old female flies used in the phagocytic ability assay		Phagocytosis RNAi knockdown (*eater* and *nimrod C1)* and overexpression *(Rab5)* in hemocytes using *Hemese (He)-Gal4, UAS-GFP.*
Le Bourg et al., 2009 [86]	*Beauveria bassiana* (Fungus) (Concentration not specified)	Hypergravity assay pretreatment before infection (flies kept at 3 or 5 g hypergravity for 2 weeks starting at 2 days of age with 20-min pauses twice a week for vial changes) Heat shock assay pretreatment before infection (flies placed at 37 °C 5 min daily for 5 successive days starting at 5 days of age) Cold Shock assay pretreatment before infection (placed at 0 °C 60 min daily for two periods of 5 days starting at 5 days of age) Climbing assay after infection (measured via vertical vial and mechanical stimulation after 20 s) Lifespan assay following infection	Flies raised at 25 °C 18-day-old separated males and females infected in the hypergravity assay pre-treatment 12-day-old separated males and females infected in the heat shock assay pre-treatment 19-, 26-, 33-, and 40-day-old separated males and females infected in in the cold shock assay pre-treatment and in the lifespan assay 3–4-week-old separated males and females infected in the climbing assay (scores measured 3 or 7 days after infection)	Suggested role for the Toll pathway	Environmental stress (hypergravity, heat shock, and cold shock effects on response to infection)
Le Bourg et al., 2011 [87]	*Beauveria bassiana* (Fungus) (Concentration not specified)	Infection survival assay with and without cold shock pre-treatment Cold shock survival assay in the absence of infection (flies kept at 0 °C for 60 min per day for two 5-day periods separated by a 2-day period without cold shock) Heat shock survival assay with and without cold shock pre-treatment (flies placed at 37 °C and observed every 5 min)	Flies raised at 25 °C 41-day-old separated males and females used in the infection survival assay with cold shock pre-treatment 1-,2-, 3-, 4-, 5-, and 6-week-old separated males and females used in the cold shock survival assay in the absence of infection 40–44-day-old separated males and females were used in the heat shock survival assay		Sexual dimorphism (cold stress increased longevity of males but not females. Cold stress increased resistance to infection in males but not in females) Age-dependent response to environmental stress (negative effect of cold stress on longevity of 5–6-week-old flies)
Le Bourg et al., 2011b [88]	*Beauveria bassiana* (Fungus) (Concentration not specified)	Cold shock assay pre-treatment (flies kept at 0 °C for 16 or 18 h overnight) Infection survival assay with and without cold shock pre-treatment Lifespan assay with and without cold shock pre-treatment Heat shock survival assay with and without cold shock pre-treatment (kept at 37 °C and observed every 5 min until dead) Climbing assay with and without cold shock pretreatment (measured via vertical vial and mechanical stimulation after 20 s)	Flies raised at 25 °C 12-day-old separated males and females used in the infection assay following cold shock pre-treatment Separated males and females used in the lifespan assay following cold shock pre-treatment (from 13 days of age until death) 13-day-old separated males and females used in the heat shock survival assay 2-, 3-, and 4-week-old separated males and females used in the climbing assay	Toll pathway (*Dif^1^* mutants used in the climbing assay, heat shock assay, lifespan assay, and infection survival assay)	Environmental Stress
Zheng et al., 2018 [89]	*Erwinia carotovora carotovora 15* (Gram-) (~6 × 10^5^ CFUs of *Ecc15)*	Infection survival assay by pricking after desiccation Desiccation assay (flies kept in empty vials without food for 2 h)	Flies raised at 25 °C 7- and 40-day-old females	Toll/IMD/NF-kB pathway (PGRP-LC, PGRP-LE, 20E)	Environmental Stress
Kubiak et al., 2017 [85]	*Beauveria bassiana* (Fungus)	1- and 4-week-old males and females	Flies raised at 25 °C Barrier Defenses Sexual Dimorphism	Injection assay (cuticle inoculation)	
